# Recent findings and applications of biomedical engineering for COVID-19 diagnosis: a critical review

**DOI:** 10.1080/21655979.2021.1987821

**Published:** 2021-10-18

**Authors:** Le Minh Bui, Huong Thi Thu Phung, Thuy-Tien Ho Thi, Vijai Singh, Rupesh Maurya, Khushal Khambhati, Chia-Ching Wu, Md Jamal Uddin, Do Minh Trung, Dinh Toi Chu

**Affiliations:** a NTT Hi-Tech Institute, Nguyen Tat Thanh University, Ho Chi Minh City, Vietnam; b Department of Biology, Faculty of Science and Technology, Universitas Airlangga, Surabaya, Indonesia; c Center for Biomedicine and Community Health, International School, Vietnam National University, Hanoi, Vietnam; d Department of Biosciences, School of Science, Indrashil University, Mehsana, Gujarat, India; e Department of Cell Biology and Anatomy, College of Medicine, National Cheng Kung University, Tainan, Taiwan; f ABEx Bio-Research Center, East Azampur, Dhaka, Bangladesh; g Graduate School of Pharmaceutical Sciences, College of Pharmacy, Ewha Womans University, Seoul, Republic of Korea; h Institute of Biomedicine and Pharmacy, Vietnam Military Medical University, Hanoi, Vietnam

**Keywords:** COVID-19, biomedical engineering, diagnosis, CRISPR, immunoassay, microfluidic devices, RT-PCR, iNAAT

## Abstract

COVID-19 is one of the most severe global health crises that humanity has ever faced. Researchers have restlessly focused on developing solutions for monitoring and tracing the viral culprit, SARS-CoV-2, as vital steps to break the chain of infection. Even though biomedical engineering (BME) is considered a rising field of medical sciences, it has demonstrated its pivotal role in nurturing the maturation of COVID-19 diagnostic technologies. Within a very short period of time, BME research applied to COVID-19 diagnosis has advanced with ever-increasing knowledge and inventions, especially in adapting available virus detection technologies into clinical practice and exploiting the power of interdisciplinary research to design novel diagnostic tools or improve the detection efficiency. To assist the development of BME in COVID-19 diagnosis, this review highlights the most recent diagnostic approaches and evaluates the potential of each research direction in the context of the pandemic.

## Introduction

In December 2019, a novel coronavirus causing a severe pneumonia disease was first detected in patients in Wuhan, China and expeditiously spread throughout the world soon after. In March 2020, the World Health Organization (WHO) declared the coronavirus disease 2019 (COVID-19) outbreak caused by the severe acute respiratory syndrome coronavirus 2 (SARS-CoV-2) as a global pandemic. Within 19 months, SARS-CoV-2 has been transmitted to almost all countries in the world and has infected more than 203 million people as of August 10^th^, 2021. The disease has been responsible for over 4 million deaths worldwide [1]. The pandemic has reduced global economic growth from −4.5 to −6.0% in 2020, with a partial recovery of 2.5% to 5.2% projected for 2021. Global trade is estimated to have fallen by 5.3% in 2020, but is projected to grow by 8.0% in 2021 [2]. COVID-19 is highly contagious and tends to easily transmit among close contacts via exposure to infectious respiratory fluids including very fine respiratory droplets and aerosol particles produced from breath, coughs, and sneezes. The extremely high transmission rate of COVID-19 has posed a high risk to the community and put enormous pressure on healthcare systems [3,4].

COVID-19 symptoms are typically high fever, dry cough, sore throat, and difficulty breathing that appear within 2–14 days after the incubation period and it may overlap with influenza or common cold. A pressing need has arisen to rapidly and accurately identify virus carriers to protect the public’s health [5]. Diagnostics play a central role in the containment of COVID-19, as it allows for identification, isolation, and contact tracing of the virus carriers, as well as rapid implementation of measures to stop the spreading of virus [6–10]. Easily missed by conventional symptom screening, however, asymptomatic people may become major virus spreaders [11]. Therefore, highly sensitive and specific SARS-CoV-2 detection methods have always been great demand [12,13].

In 2020, the global market for COVID-19 diagnostic services was valued at $60.3 billion and in 2021 it was predicted to reach $84.4 billion and $195.1 billion by 2027 [14]. There are various methods for detecting COVID-19, including immunoassays, protein assays, viral plaque assays, hemagglutination assays, viral flow cytometry, etc [15–18]. Conventionally, the majority of pathogen diagnostics have been developed for laboratory-based detection, including immunoassay-based and reverse transcriptase-polymerase chain reaction (RT-PCR)-based methods [19]. However, the time-consuming conventional diagnostic procedures were soon overwhelmed by the unmatched rate of infection and hospitalization. Therefore, more and more point-of-care testing (POCT) and rapid methods have been exploited for supporting the medical decision-making process or self-health monitoring, including isothermal nucleic acid amplification technique (iNAAT) [20], clustered regularly interspaced short palindromic repeats (CRISPR)-based methods [21–23], biosensors, and microfluidic devices [24]. Automated artificial intelligence models have also been proposed to facilitate high-throughput and consistent diagnosis. These technologies have been developed rapidly while the pandemic is going on. Here, we highlight the most recent progress of these biomedical engineering (BME) technologies applied to COVID-19 diagnosis in this review, as well as provide insights into how the research direction in this field has shifted in response to practical demands for disease surveillance and personal healthcare.

## Laboratory-based immunoassay methods

**Figure 1.**
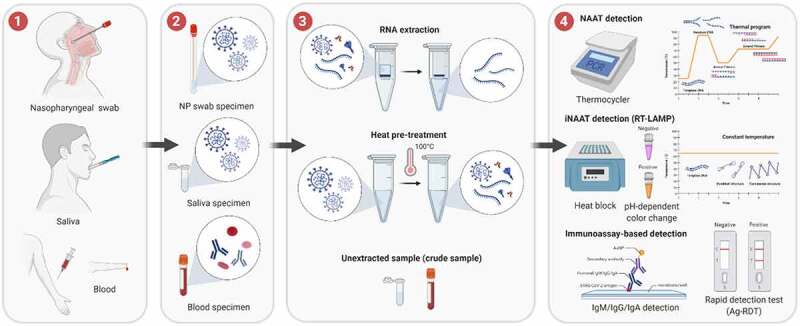
General COVID-19 diagnostic workflow using molecular testing (NAAT, iNAAT and immunoassay-based detection). (1) Sample collection methods; (2) Types of samples; (3) Sample processing or pre-treatment; (4) Test reaction and result reading. The methods illustrated are the most commonly used for COVID-19 diagnosis and the alternatives in each step are mostly interchangeable, except that blood samples are not used for NAAT and iNAAT techniques, and extracted RNA is not used for immunoassay detection. The image was created with BioRender.com

SARS-CoV-2 infection stimulates the humoral immune system to produce specific antibodies, including immunoglobulin A, M, and G (IgA, IgM, IgG) [25–27] that appear in patient blood by specific kinetics. This information has guided the development of immunoassays, which are mainly enzyme-linked immunosorbent assay (ELISA) and chemiluminescent immunoassay (CLIA), for SARS-CoV-2 detection, tracing, and seroprevalence studies (Figure 1).

ELISA was commonly used at the early stage of the pandemic as a qualitative or semi-quantitative method to detect humoral anti-SARS-CoV-2 immunoglobulins or virus antigens. It uses an immobilized SARS-CoV-2 antigen (Ag) to capture its cognate humoral antibody (Ab). The Ag-Ab binding is usually detected by a secondary Ab that was labeled with an enzyme (normally horseradish peroxidase, HRP) to catalyze a color change reaction. The titer of host immunoglobulins or virus antigens can be determined (indirect ELISA and direct ELISA, respectively) by measuring the colorimetric changes on a microplate reader. Among the initial attempts, Amanat *et al*. used purified recombinant S protein (with modifications) and its receptor-binding domain (RBD) to develop an indirect ELISA to detect IgM and IgG in serum, revealing the strongest binding reactivity for the full-length S protein and the correlation between ELISA titers and virus neutralization [28]. Peterhoff *et al*. developed an ELISA using SARS-CoV-2 RBD as the antibody-catching Ag to achieve high specificity (92–98%) and high specificity (99.3%) for IgA, IgG, and IgM in the serum at > 10 days after PCR positive [29]. RBD ELISA for testing IgG was found to be sufficient for COVID-19 diagnosis with high sensitivity and specificity (88% and 98%, respectively) [30]. Since IgM positive predictive value (PPV) was insufficient, IgA specificity was low, and their presence in the blood was short-lived [31–33], both IgM and IgA are not reliable markers for ELISA. However, combined detection of IgA/IgM/IgG was suggested as the most sensitive assay to detect SARS-CoV-2 31. Interestingly, Kyosei *et al*. proposed a *de novo* system for SARS-CoV-2 antigen detection by coupling a spike protein (S1)-detecting sandwich ELISA system with thio-nicotinamide adenine dinucleotide (thio-NAD) cycling. By adding 10 min of thio-NAD cycling to the ELISA procedure and measuring S1 concentration using a plate reader at OD_405_^,^ high sensitivity of 10^4^ viruses per reaction can be achieved [34].

ELISA can be used in low-cost settings. It is easy to perform and automated, but it is time consuming process (2–5 h) and it can be easily contaminated. That makes another alternative, CLIA, more suitable when a faster turn-around time (1–2 h) is required. Similar to ELISA, but instead of using an enzyme, CLIA uses a luminophore to conjugate the secondary Ab, so that the specific Ag-Ab binding will trigger a light or fluorescent emission. A few studies found that ELISA sensitivity is similar or slightly better than that of CLIA in detecting humoral Ag or viral Ab [35,36]. Ma *et al*. used highly purified RBD to make a set of CLIA kits for detecting RBD-specific IgA/IgM/IgG, reaching 96.8–98.6% sensitivity and 92.3–99.8% specificity, also combined the IgA/IgG detection kits to boost the sensitivity and specificity to 99.1% and 100%, respectively [37]. For both ELISA and CLIA, automated, high-throughput detection systems, such as Diazyme DZ-Lite 3000 Plus (Diazyme Laboratories, USA) [38], or MAGLUMI CLIA (Snibe, China) [39] have demonstrated high sensitivity, specificity, and the capacity to process multiple samples simultaneously. It is noteworthy that a wide variety of performance between commercially available ELISA/CLIA kits was found [40–44], thus the need of validating the assays before use is very critical. Wu *et al*. showed that the combination of antibody detection and existing RT-PCR greatly enhanced SARS-CoV-2 detection, from 48.1% (RT-PCR alone) to 72.2% [45]. Based on the process of SARS-CoV-2 infection and the production of specific antibody responses, a diagnostic IgG and IgM laboratory-based immunoassay would be the most effective method for COVID-19 diagnosis.

## Rapid detection tests (RDTs) for POCT

Even though sharing the same working principle as ELISA, an RDT is formatted into a portable cassette or dipstick to perform the test at POC or home (Figure 1). SARS-CoV-2 RDTs can indirectly detect the virus through humoral antibodies (IgM, IgG, IgA), referred to as Ab-RDTs, or directly detect a surface antigen of the virus, referred to as Ag-RDTs. For convenient result reading, the RDT is engineered as a later flow immunoassay (LFA) device, comprised of a nitrocellulose membrane contained in a plastic housing, immobilized Ab, and labeled Ag/Ab (usually conjugated with colloidal gold). The presence of a target molecule is indicated by a color band that appears on the test line. RDT kits are inexpensive, very simple to use without training or laboratory equipment, and usually have a short time-to-result of 10–15 minutes. Therefore, RDT has become one of the most widely used methods for SARS-CoV-2 detection, especially for POCT screening and personal use. However, their uses in clinical diagnosis are limited to certain circumstances, mostly depending on the stage of disease progress, viral loads, and viral prevalence [46].

A systematic meta-analysis by Ghaffari *et al*. on 62 commercially available serological (antibody detecting) test kits (ELISA, CLIA, RDT) revealed a wide range of sensitivity variation (almost 0% to 100%), while most of the kits exhibited >90% specificity [47]. Noticeably, most of the worst performance was from RDT kits. It was also confirmed that these serological kits are effective in later periods of the disease progression [47]. From meta-analysis, Bastos *et al*. found that the overall sensitivity of serological immunoassays was significantly higher at least 3 weeks from the illness onset (69.9–98.9%) as compared to the results from the first week (13.4–50.3%) [48]. Even though these serological detection kits are not sufficiently sensitive at the early stage of infection, they are important tools to investigate one’s past infection [28] and provide information on how the virus spreads in a community [49].

Ag-RDT kits are designed the same way as Ab-RDT kits, except that the targets are viral surface proteins and the virus is directly detected. Ag-RDT assays were shown to be better screening tools than Ab-RDT kits [50]. However, the performance of commercial Ag-RDT kits can be vastly different [51–53]. A clinical evaluation of 122 SARS-CoV-2 Ag-RDT kits with the European Conformity (CE) mark reported a wide range of performance variation that 78.7% of the kits exhibited a sensibility >75% on samples with high viral loads, and 19.8% of the kits showed a sensitivity >75% for medium viral loads [54]. With qRT-PCR as the gold standard, Ag-RDT sensitivity was significantly different between the symptomatic (80–96.52%) and asymptomatic group (37–71.43%) [50,55,56], but its positive predictive value (PPV) was higher in agreement with viral cultivability [57] and its sensitivity was only slightly lower than the qRT-PCR as long as the virus isolated from the sample that was cultivable [58]. The sensitivity of an Ag-RDT kit was found to be dramatically reduced from 86.5% to 53.8% after 7 days of illness onset [59]. Since low viral loads (Ct > 30) are linked to low viral culture positivity or infectivity [60,61], the proper use of Ag-RDT kits is to detect infectious cases. Nevertheless, for screening mixed symptomatic and asymptomatic groups, serial testing with the minimal 3-day interval of between tests can also increase the sensitivity of Ag-RDT to over 98% [58]. Also, for community screening, the short sample-to-answer time and the repeat testing of Ag-RDTs were demonstrated to be more important than the sensitivity [62].

## Nucleic acid amplification testing (NAAT) methods

NAAT, or specifically, quantitative RT-PCR (qRT-PCR) was among the earliest diagnostic tools developed for the detection of SARS-CoV-2 from the available sequence data shared from China (Figure 1). A few days after the initial SARS-CoV-2 outbreak, a full genomic sequence of the virus isolated from a patient from Wuhan was released and deposited on GenBank (accession number MN908947.3). It was the first genomic data for the design of primers for qRT-PCR by researchers from China, France, the USA, Japan, Germany, Hong Kong, and Thailand. These protocols were later compiled and made available online through WHO [63]. In order to facilitate the rapid sharing of SARS-CoV-2 sequences, a data-sharing service hosted by the Global Initiative on Sharing All Influenza Data (GISAID) was introduced (https://www.epicov.org). Numerous efforts have been made by scientists worldwide to optimize qRT-PCR procedures and produce commercial SARS-CoV-2 diagnostic kits to support disease surveillance at hospitals, healthcare centers, and in the community.

As instructed by the protocols published in the WHO guideline, various SARS-CoV-2 genomic targets, including structural genes, *N* (nucleocapsid), *RdRp* (RNA polymerase), *S* (spike protein), *E* (envelope protein), *Orf1ab* (replication complex), and a non-structural gene, *nsp14* [63], were used for the amplification. According to Corman *et al*., qRT-PCR protocols with the *E* gene or *RdRp* gene were shown to produce the best results with a limit of detection (LOD) of 3.2 to 5.2 RNA copies per reaction [64]. A comparative study was also reported the high analytical sensitivity of qRT-PCR using Corman E gene and CDC N1 primer-probe sets (LOD = 6 RNA copies per reaction) [65]. However, quickly after the initial outbreak, growing evidence showed that the mutations occurred in the SARS-CoV-2 genome were prone to significantly reducing the sensitivity of available qRT-PCR procedures [66–69]. Based on 31,421 genomic sequences shared on GISAID as of July 23^rd^, 2020, Wang *et al*. found that virtually all the recommended primer sites have undergone mutations and the *N* gene primers and probes covered most of the mutated spots [70]. Later evidence of mutations of *E* and *N* genes hinted at the escape of the virus from qRT-PCR detections [71–73]. Interestingly, the most common mutation was found to be cytosine-to-uracil type, which was caused by a strong mRNA editing mechanism catalyzed by apolipoprotein B mRNA-editing enzyme, catalytic polypeptide-like (APOBEC) cytidine deaminase during its involvement in the innate immune host response [74,75]. These findings emphasized the need of developing more multiplex assays for COVID-19 diagnosis. As the first commercial multiplex qRT-PCR for SARS-CoV-2, QIAstat-SARS, while targeting both *E* and *RdRp* genes, achieved a LOD of 1 RNA copy per µl and very high percent agreement (97%) with WHO RT-PCR assay [76]. Moreover, *in silico* analysis of PCR performance with known virus variants was highly recommended for proper adjustments of the optimal cycle threshold depending on the changes in the amplification curve [77]. When the co-infection of SARS-CoV-2 and influenza viruses has become more frequent and increase the risk of severity and mortality of COVID-19 patients [78], multiplex qRT-PCR assays for the simultaneous detection of SARS-CoV-2 and influenza A/B also become necessary [79,80].

Studies have identified the presence of SARS-CoV-2 in the respiratory tract (sputum, nose, bronchoalveolar lavage fluid (BALF) [81], nasopharynx and oropharynx [82], etc.), gastrointestinal tract (stool, anal swab [83], etc.), even in the retina [84], olfactory mucosa [85] and brain [86] of COVID-19 patients. However, only a limited number of specimen types can be used for qRT-PCR detection. Based on the studies on qRT-PCR sensitivity and specificity varied in specimen type, nasopharyngeal (NP) swab has widely been used upper respiratory tract specimen, sputum is for the lower respiratory tract sampling [81,, 87,, 88], while oropharyngeal (OP) swab was not recommended due to its lower positive rate. However, the disadvantage of using NP swabs is the discomfort of the testes and the risk of complications, such as broken swabs or nasal bleeding [89]. Alternatively, a combined nasal/OP swab can be used to provide excellent sensitivity while releasing the stress of NP swab shortages [90]. Later recognized, but with the high sensitivity and specificity of saliva-based qRT-PCR (84.2–95.2% and 98.9%, respectively), saliva has become an appealing noninvasive alternative to NP swabs because it is easy and painless for self-sampling, child-friendly, and safer for healthcare workers [91–95]. It was even proposed as the gold-standard sample for COVID-19 diagnosis [96] and it was shown practically to perform similarly to NP swab-based RT-PCR [97].

Even though qRT-PCR is usually considered the gold standard for COVID-19 diagnosis, it has shown several critical limitations in practice as the results of some pre-analytical and analytical vulnerabilities, including erroneous sampling, low assay accuracy, unaware mutations, lack of understanding of viral load kinetics [98,99]. In early reports, the immature development of NAAT technology for SARS-CoV-2 detection can be blamed for the moderate clinical sensitivity of qRT-PCR assays (71–82.2%) [100–103]. However, after more than a year of intensive development and optimization, the clinical performance of qRT-PCR has not been markedly improved [104], especially for screening in population-based and hospital settings [105,106].

Modifications to the qRT-PCR procedure have been proposed and demonstrated to improve the overall capacity, reduce turn-around time, cost, or adapt the system to POCT settings, such as employing patient-collected swabs and saline gargles [107] or saliva [108,, 109], unextracted clinical samples [110–114], or portable miniature PCR workstations [115–117]. Noticeably, a novel approach to PCR, namely viability RT-qPCR, employed platinum chloride to treat NP swab samples and prevent the amplification of SARS-CoV-2 RNA in free form or from virions with damaged capsids, thus detecting the only RNA associated with intact virions, indicating infectivity [118]. This method is a suitable tool to ascertain one’s infectiousness without the need to perform virus culture, avoid false positives caused by contaminated RNA from the environment and identify noninfectious, prolonged RNA shedding from patients.

Strategy-wise, pooling or group testing was also suggested as an attractive low-cost tactic to screen a large population with low virus prevalence, preserving 69–93% of the cost without reducing the detection efficiency [119,120]. Alternatively, serial testing has been demonstrated to be effective in improving the clinical sensitivity of qRT-PCR to above 90% [58,100,103]. The remaining challenge to qRT-PCR is the limited capacity to precisely process a large number of samples simultaneously [98]. While automated, high-throughput qRT-PCR systems such as cobas 6800/8800 (Roche Molecular Systems, USA) [121], Alinity m2000 (Abbott Molecular, USA) [122], GeneXpert Xpress (Cepheid, USA) [123], etc., can partly resolve the analytical and capacity problem [124], they cannot help reduce human-related errors in pre-analytical steps [98]. Also, most of these systems either require high investment or have low-throughput capacity. Therefore, further improvements are still needed to adapt PCR to the pandemic circumstances.

## Isothermal nucleic acid amplification testing (iNAAT) methods

iNAATs are alternatives to conventional PCR and are usually designed for POCT diagnosis, in which the nucleic acid amplification is performed at a constant temperature by avoiding thermal denaturing of the double-strand DNA (dsDNA) template (Figure 1). Among the iNAAT methods, loop-mediated isothermal amplification (LAMP), developed by Notomi *et al*. (2000) [125], has been the most frequently used one. This method utilizes a DNA polymerase with strand displacement activity (usually Bst DNA polymerase) and 4–6 primers that recognize 6–8 distinct regions on the target DNA sequence. The whole process requires only incubation at 60–65°C for less than 1 hour, producing 10^9^ copies of a target sequence. Amplified products can be conveniently visualized with various dyes, such as phenol red, hydroxy naphthol blue, leuco crystal violet (LCV), SyBr Green, or by coupling the reaction with an LFA strip. The addition of reverse transcriptase to the LAMP assay (RT-LAMP) allowed for the detection of viral RNA at LOD of 5–10 copies per reaction, even without RNA extraction [126]. Most reports achieved the clinical sensitivity and specificity of RT-LAMP within 75–100% and 98.7–100%, respectively, while the LOD ranged from 1 to 304 copies per reaction [127–134]. Another advantage that makes LAMP fit for POCT is the use of lyophilized reagents without sacrificing quality [135], which expands the kit shelf-life to years at 4°C or several weeks at room temperature [136,137]. Nevertheless, LAMP performance is heavily dependent on its custom design and might not yet be comparable to qRT-PCR in some cases, as it was reported to be reliable up to the viral load equivalent of Ct (cycle threshold) < 30 [131], which was in line with the observations from other groups [138,139].

Another iNAAT option for SARS-CoV-2 diagnosis is recombinase polymerase amplification (RPA), which was invented by Piepenburg *et al*. [140]. In this method, Bsu DNA polymerase I (large fragment) is used to extend the 3′ termini of two oligonucleotides (primers). The strand hybridization of primer-ssDNA is mediated by a recombinase (T4 uvsX) and other proteins (T4 gp32, T4 uvsY). RPA normally operates at 37–42°C and takes only 10 minutes to complete the amplification. The fast process, the smaller number of primers required, the low working temperature, and the versatility of targeting multiple sequences simultaneously have made RPA an excellent alternative to PCR and LAMP. Based on RT-RPA, Xia *et al*. introduced an one-pot, 30-min WEPEAR (whole-course encapsulated procedure for exponential amplification from RNA) procedure for simultaneously detecting N and S genes of SARS-CoV-2 at the LOD of 1 RNA copy per reaction [141]. A clinical evaluation of RT-RPA for SARS-CoV-2 detection showed the sensitivity, specificity, and LOD of 98%, 100%, and 7.7 RNA copies/µl, respectively, which was comparable to qRT-PCR (5 copies/µl) [142].

Nicking enzyme-assisted amplification reaction (NEAR), or nicking enzyme-assisted amplification (NEAA) relies on a nicking endonuclease (NE, such as Nt.BstNBI, Nb.BtsI, and Nb.BsrDI), in addition to a strand-displacing DNA polymerase (Bst DNA polymerase) [143]. NEAR circumvents the need for a thermal denaturing dsDNA template by using NE to recognize a specific dsDNA sequence covered by the primer region and introduce a nick site on one strand, exposing its 3′ end for elongation. A typical NEAR takes 15–30 minutes at 54–58°C to complete and is extremely efficient in target amplification. However, NEAR is not as popular as LAMP or RPA due to its tendency to produce nonspecific products [144]. Despite that disadvantage, NEAR was soon adopted into a commercial diagnostic tool, the ID Now™ system (Abbott, USA), in which various diseases can be detected within 5 minutes directly from clinical samples. Even though ID Now™ is widely used in the USA, contradicting evaluations of its performance for SARS-CoV-2 diagnosis have been reported. Most clinical reports showed 54.8–94% positive agreement between ID Now™ and qRT-PCR based platforms [145–150] and some performance may be caused by errors in specimen preparation or improper handling of the machine. In addition, Tu *et al*. reported that the high diagnostic value from this system can be achieved with symptomatic patients [151].

The diagnostic value of iNAATs is usually compared to qRT-PCR or other conventional diagnostic methods, thus it is difficult to justify the relative performance of iNAATs to each other. So far, only a few studies have directly compared iNAATs for detecting a specific target. Tran *et al*. found that RT-LAMP is superior to the other two iNAATs that utilize Bst DNA polymerase for detecting SARS-CoV-2, cross-priming amplification (CPA), and polymerase spiral reaction (PSR) with a 20–40 times lower LOD value [135]. Naveen and colleagues showed that the LOD of RT-LAMP was equal or one order of magnitude lower than that of RT-RPA in detecting two ginger-infecting viruses [152] and cardamom vein clearing virus [153]. These data support the conclusion that LAMP is currently the most suitable iNAAT for SARS-CoV-2 diagnosis. With the recent demonstrations of using alternative specimens, including saliva [154,155], and exhaled breath samples (by a face mask-based collector) [156], RT-LAMP has been transformed to adapt better to the POC diagnostic settings.

## CRISPR-based diagnostics

CRISPR and CRISPR-associated proteins (Cas) systems are prokaryotic RNA-mediated immune systems that prevent bacteriophage infection and plasmid transfer [157–159]. CRISPR is divided into two classes, Class 1, which includes groups I, III, IV, and Class 2, which includes groups II, V, and VI, and further categorized into more than 30 subgroups [160]. In which, Cas9 (formerly Csn1) represents subgroup II-A and is the most widely used Cas nuclease for genome editing in a wide range of organisms and cell types [161–164]. The method has been used as an antimicrobial agent for the removal of bacterial pathogens [165–167] and viruses including HIV-1 [168,169], human papillomavirus [170], hepatitis B virus [170,171], and SARS-Cov-2 [172]. In Class 2, there is also a nuclease in the VA subgroup, Cas12a (formerly Cpf1) isolated from *Francisella novicida*. It has a different mechanism of action than Cas9, with the ability to use a single crRNA molecule to find the target sequence and cut the target sequence at two staggering sites. In addition, Cas12a also exhibits collateral nuclease/cleavage activity, which is capable of cutting nonspecific single-stranded DNA fragments immediately upon binding to the target sequence [173]. These features make Cas12a a more favorable tool for application in the specific detection of DNA/RNA sequences.

**Figure 2.**
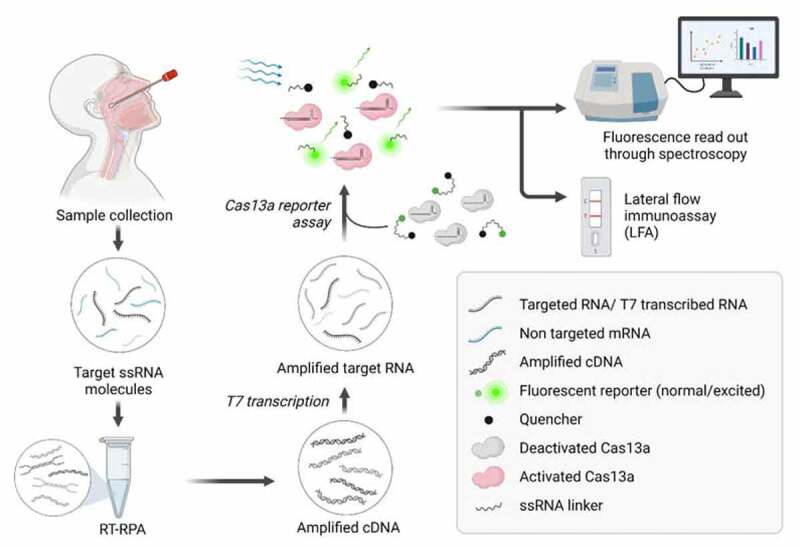
CRISPR-Cas13a based detection of SARS-CoV-2. Nasopharyngeal and oropharyngeal specimens are collected via sterile swabs. The collected sample is then diluted in an appropriate buffer, followed by a few heating steps. Sample heating steps release ssRNA from the virus and facilitate the deactivation of nuclease if any is present in the sample. Following the heating step, the viral RNA is subjected to RT-RPA for the amplification of target sequences in the form of cDNAs, which are in turn transcribed by T7 RNA polymerase. The accumulated amplification products of the targeted RNA sequence are provided for Cas13a-based detection assay. Cas13a recognizes T7-transcribed RNA sequences if appropriate guide RNA (gRNA) is presented. This leads to the activation of Cas13a and displays its nonspecific RNAase activity, resulting in the nonspecific cleavage of the fluorophore-ssRNA-quencher complex. The florescence emitted by the fluorophore can be quantified via spectroscopy, indicating the concentration of the ssRNA template. Alternatively, the cleaved reporter molecule can be detected via paper LFA. The image was created with BioRender.com

Other types of Cas nucleases are also beginning to be exploited for nucleic acid detection purposes, including Cas13a (formerly known as C2c2, belonging to subgroup VI-A) and Cas13b (formerly known as C2c6, belonging to group VI-B1) [174]. With their ability to recognize RNA, Cas13a and Cas13b were used for the first time in RNA editing [175,176]. Using their nonspecific cleavage RNAse activity of single-stranded RNA, these two nucleases and Cas12a were used respectively in the nucleic acid detection kits including Specific High Sensitivity Enzymatic Reporter UnLOCKing (SHERLOCK) [177], SHERLOCK v2 [178] and DNA endonuclease-targeted CRISPR trans reporter (DETECTR) [173]. So far, SHERLOCK has been reported to be able to detect different pathogens at ng or pg concentration of DNA or RNA, such as ZIKA virus with a titer as low as 2.1 attomolar (aM) from clinical samples containing *Escherichia coli* and *Pseudomonas aeruginosa* [179]. Additionally, the CRISPR-Cas13a-based system was shown to identify single nucleotide polymorphisms in humans as well as to discriminate between the antibiotic-resistant strains of *Klebsiella pneumoniae* with high specificity [179]. SHERLOCK v2 was developed for multiplex detection of nucleic acid in a single reaction chamber at a concentration range of attomolar (aM) of the target. Integrating SHERLOCK v2 signal amplification with LFA, the SHERLOCK v2 paper-based test can detect as low as 2 aM of a nucleic acid target (acyltransferase gene) after 90 min, with less background and increased signal intensity [21]. The SHERLOCK system was further modified into miSHERLOCK (minimally instrumented SHERLOCK) as a low-cost, self-contained, POCT device that used crude saliva samples and required less than 1 hour of sample-to-answer time [180]. Despite the excellent efficiency, the number of reports available for CRISPR-based diagnostics (CRISPR-Dx) for viruses, bacteria, mutations, and SNPs is still limited [181]. Figure 2 depicts the workflow for real-time CRISPR-Cas13a based detection of SARS-CoV-2 from clinical samples.

Broughton *et al*. developed a CRISPR-Cas12-based LFA for the detection of SARS-CoV-2 in less than 40 min [182]. In this work, they tested 36 patients of SARS-CoV-2 and 42 patients with other respiratory infections. It was found that CRISPR-Cas12-based performed on par with the RT-PCR assay as it reached 100% positive and negative predictive agreement. Similarly, Ding *et al*. developed an All-In-One Dual CRISPR-Cas12a (AIOD-CRISPR) assay for the detection of SARS-CoV-2 [182]. They targeted the nucleoprotein encoding gene and found the results were consistent with the RT-PCR assay. This CRISPR-based tool was inexpensive to produce and required only 20 minutes of time-to-result using clinical samples [182]. In another attempt, Chen *et al*. coupled LAMP and CRISPR-Cas12a for the rapid diagnostic of SARS-CoV-2 [183]. With the help of smart phone and 3-D printing equipment, the virus has been detected by the naked eye, which was a great advantage for POCT. RNA of SARS-CoV-2 has been detected within 40 minutes, with a high sensitivity of 20 SARS-CoV-2 RNA copies per sample. Additionally, Huang *et al*. developed a CRISPR-Cas12a-gRNA complex and fluorescent probes to detect nucleic acid produced by RT-PCR or RT-RPA [184]. It was found that with the aid of CRISPR-Cas12 system, SARS-CoV-2 was detected within 50 minutes, with the LOD of 2 copies per nasal swab. More recently, Li *et al*. established a CRISPR-based LFA for POCT of SARS-CoV-2 that can detect 10–100 virus RNA copies/μL from clinical samples [185]. The system was further improved by developing easy-readout and sensitive enhanced (ERASE) strips to reach a LOD of 1 copy/μL. The method was then used for testing 649 clinical samples, achieving 90.67% positive predictive agreement and 99.21% negative predictive agreement. Similarly, Yang *et al*. used Cas13a to couple with a universal autonomous enzyme-free hybridization chain reaction (HCR) by designing a cleavage reporter assay [186]. Once Cas13a found target sequences, it triggered the downstream HCR circuits. They designed three guide RNAs (gRNAs) for targeting SARS-CoV-2 *S, N*, and *Orf1ab* genes and succeeded in detecting the target sequences within 1 h at attomolar level sensitivity [186]. Even though CRISPR systems are mainly used for genome editing, this growing evidence has demonstrated their value in boosting the performance of iNAAT detections, making iNAATs more suitable to POC settings.

## Microfluidic devices and biosensors for SARS-CoV-2 diagnostics

Microfluidics is an exponentially growing field of engineering and has shown a rather large number of applications in a wide range of areas like rapid diagnostics, biomedical therapy, organ culture, 3D culture, *in vitro* toxicity testing, nucleic acid extraction, and amplification, drug delivery, single-cell analysis, and many more [171,187–192]. This technique is based on the precise manipulations of micro-scale fluids in micro-channels. It has been widely used and have shown number of distinctive advantages, including rapid sample processing, assay controllability, portability, millimeter-scale design, multi-tasking capability, low-volume assay, and low-cost requirements, in comparison to other conventional platforms. Particularly, microfluidic devices have demonstrated high practical and diagnostic values in the field of rapid, POC pathogen detection, such as assays targeting parasites and viruses [193–196].

Isolation of nucleic acids is the critical step in a NAAT/iNAAT workflow, but can also be time-consuming, costly, and tedious. The product quality and efficiency of the isolation step can be inconsistent between batches or labs. Therefore, as aforementioned, automatic, high-throughput extraction and detection devices can facilitate the whole diagnostic procedure, from obtaining the clinical sample to reading results. Brassard *et al*. designed a microfluidic device for the extraction of DNA from blood samples which helped reduce the time and chemical expense for the extraction [197]. Similarly, Geissler *et al*. established a microfluidic device for performing the whole process of bacteria identification for *E. coli* O157:H7 from cell lysis, multiplex PCR amplification, to on-chip hybridization with fluorescent gene markers [198]. More recently, Sullivan *et al*. [199] designed microfluidic devices for the purification of nucleic acids directly from blood samples using isotachophoresis (ITP), which was directly used for POCTs. Qiu *et al*. introduced a fully disposable heat capillary tube without an electric supply for DNA amplification, in which PCR reagents were repeatedly passed through different temperature zones [200]. The device allowed a single-step nucleic acid dipstick assay for visualizing DNA amplification by the naked eye. It achieved the sensitivity of 1.0 TCID_50_/mL for detecting H1N1 within 35 minutes and was suitable for instrument-free diagnosis in remote areas [200].

Under the burden of COVID-19 pandemic, the combination of microfluidics and the available diagnostic methods has provided timely upgrades to the available diagnostic procedures. A semi-automatic high-throughput microfluidic device was developed for measuring in parallel the anti-SARS-CoV-2 IgG/IgM levels in 50 serum samples and achieved a sensitivity of 95% with a specificity of 91% [201]. An Opto-microfluidic sensing platform based on gold nanospikes was developed for the detection of antibodies in 1 μL of human plasma within 30 minutes. This label-free platform reached a relatively low LOD of 0.08 ng/mL for serological testing of anti-SARS-CoV-2 antibodies presented in diluted blood plasma samples [202]. Another highly sensitive and specific portable microfluidic immunoassay system was engineered for on-site and simultaneous detection of IgG/IgM/Antigen of SARS-CoV-2 within 15 minutes [203]. Lately, Ramachandran *et al*. designed an electric field-driven microfluidic device for CRISPR-based detection of SARS-CoV-2 within 35 minutes from contrived and clinical nasopharyngeal swab samples [204].

Besides microfluidic devices, an urgent need has been arisen for POC diagnosis of COVID-19 that has motivated the invention of a portable, low-cost biosensors, especially electrochemical immunosensors. Mavrikou *et al*. utilized membrane-engineered mammalian cells electroinserted with SARS-CoV-2 Spike S1 antibody to detect the binding of SARS-CoV-2 onto the membrane via measuring changes in membrane potential [205]. The results were obtained within 3 minutes with 93% accuracy as compared to RT-PCR [205]. SARS-CoV-2 nucleocapsid protein (N) can be alternatively detected by its cognate antibody grafted on a gold-coated microcantilever surface at the LOD of 100 viral copies/mL or 0.71 ng/ml [206]. A portable, disposable electrochemical sensor made from molecularly imprinted polymers (MIPs) was capable of detecting SARS-CoV-2 N protein with a LOD of 15 fM [207]. Relying on changes in the volatile organic compounds (VOCs) in exhaled human breath to indicate SARS-CoV-2 presence, a portable electronic nose (GeNose C19) was fabricated with a metal oxide semiconductor gas sensor array and supported by machine learning models to detect SARS-CoV-2, up to a sensitivity and specificity of 86–94% and 88–95%, respectively [208].

So far, graphene has been demonstrated to be an excellent material for developing biosensors, which has shown its high conductivity, stability, and specific surface area. However, due to its lack of reactive chemical groups, it is usually functionalized by nanoparticles. For example, in order to develop LEAD (Low-cost Electrochemical Advanced Diagnostic) system, Lima and colleagues first treated a graphite pencil electrode (GPE) with glutaraldehyde solution, then coated GPE with AuNPs functionalized with cys, and finally mixed it with a solution consisting of N-(3-dimethylaminopropyl)-N-ethylcarbodiimide hydrochloride (EDC), N-hydroxysuccinimide (NHS), and human angiotensin-converting enzyme 2 (ACE2) receptor, enabling the immobilization of ACE2 on GPE surface. This device can directly capture SARS-CoV-2 in clinical samples (saliva and NP swab stored in VTM) and detect at least 229 fg/ml of S protein by measuring the signal suppression of a redox probe [Fe(CN)_6_]^−3/−4^ upon S protein – ACE2 binding [209]. It can be manufactured for only $1.5, requires 6.5 minutes of sample-to-answer time, and displays 100% sensitivity and specificity using saliva specimen [209]. Alternatively, Nguyen *et al*. functionalized graphene with 1-pyrenebutyric acid N-hydroxysuccinimide ester (PBASE) for immobilizing anti-SARS-CoV-2 spike RBD antibody [210]. SARS-CoV-2 in artificial saliva, down to 3.75 fg/ml, was recognized upon binding to the immobilized antibody by observing changes in graphene’s phononic response via Raman spectroscopy [210]. A crumpled graphene field-effect transistor (FET)-based biosensor immobilized with N- and S-protein antibodies was shown to detect N and S proteins at extremely low LOD (1 aM), surpassing ELISA sensitivity [211].

Other than electrochemical immunosensors, a SARS-CoV-2 biosensor can be integrated with microfluidic devices and iNAAT technologies as well. A face-mask-integrated SARS-CoV-2 sensor was made to collect breath-generating viruses accumulated under the mask and detect their RNAs by activating lyophilized Cas12a SHERLOCK reagents that has embedded on a paper-based microfluidic device [212]. This portable, personal testing device inherited from previous discoveries on breath sampling technologies, paper-based biosensor, and LFA for visualized monitoring of the results, allowing for a LOD of 500 IVT (*in vitro* transcribed) RNA copies per reaction [212]. Altogether, microfluidic devices, and biosensors have shown a great potential in adapting lab-based pathogen diagnostics to POC and low-cost settings while maintaining detection efficiency. However, most of these products and procedures have not been validated in a large-scale clinical trials to confirm their practical uses.

## Artificial intelligence-assisted diagnostics

The ability to make fast and accurate decisions has been a vital factor affecting the capacity of COVID-19 diagnostic systems to cope with extremely high testing volumes. With limited clinical sensitivity of qRT-PCR demonstrated at the initial stage of the pandemic, chest computed tomography (CT) and chest X-ray (CXR) was shown to efficiently support qRT-PCR and improve the overall accuracy. Compared to PCR, chest CT is easy to perform, faster, more standardized, and consistent as most of the COVID-19 patients exhibit typical radiographic features, including ground-glass opacity (GGO), crazy-paving pattern, pleural effusions, and consolidation [213]. Moreover, a chest CT scan can be used to assess the severity of symptomatic patients [214]. However, chest CT has a major drawback of relatively low specificity (25–80%) [215], causing misinterpretation of the infections caused by other pathogens, and thus cannot be used as a ground truth. The use of artificial intelligence (AI) is a promising approach to solve this problem, reducing the workload for radiologists, and improving the overall accuracy of radiography-based diagnosis.

An online processing strategy was exploited by Saba’s group by developing six models (two machine learning (ML) models, two transfer learning (TL) models, and two deep learning (DL) models) for classifying COVID-19 (CoP) and non-COVID-19 pneumonia (NCoP). They demonstrated 74.58–99.63% accuracy and 0.74–0.99 AUCs (areas under the ROC curve) with less than 2 s of inference time [216]. Another online server can distinguish COVID-19 patients from bacterial pneumonia patients and healthy people with a recall (sensitivity) of 93% and PPV of 86% while extracting main lesion features such as GGO for assisting doctor decision [217].

Due to the limited number of annotated radiographs, transfer learning techniques has been used to accelerate the training time and allow for training deep CNN networks with relatively small datasets [218,219]. Noticeably, Abbas *et al*. developed *DeTraC* (Decompose, Transfer, and Compose), a deep CNN architecture using transfer learning and class decomposition, to achieve high accuracy and specificity of 98.23% and 96.34%, respectively, with an ImageNet pre-trained CNN model (VGG19) [220]. Transfer learning is extremely beneficial for training small datasets, but when there are many positive cases to collect radiographs, pre-training on ImageNet will not be useful.

In order to develop an automatic COVID-19 prediction model, Chen *et al*. were able to prospectively collect 46,096 anonymous CT images of 106 COVID-19 inpatients for training using Unet++ [221]. The validation tests on an external dataset achieved a sensitivity and specificity of 98% and 94%, respectively, showing that the DL model performance was on par with expert radiologists and helped reduce the reading time of radiologists by 65% [221]. Shan *et al*. approached the limitations of the chest CT-based diagnosis procedure differently by building a DL-based automatic segmentation tool to quantify infection volume, dramatically reducing the image delineation time from 1–5 h to 4 minutes while achieving 91.6% Dice coefficient with the manual segmentation [222].

Other than medical computer vision, AI also provides an excellent tool for tele diagnosis of COVID-19 via examining cough and breath sounds. An AI developed by Laguarta’s group can identify asymptomatic COVID-19 patients with 100% sensitivity and 83.2% specificity [223]. Several crowdsourced annotated datasets of cough sounds are available to support research in this field, such as COUGHVID with over 25,000 recordings [224] and Coswara with recordings from 941 participants [225].

Even though the performance data encourage the use of AI in assisting COVID-19 diagnosis, it still needs lots of effort for realization in clinical practice. It is not just a matter of accuracy to gain trust from clinicians, especially in the life-death decision-making process. Therefore, diagnostic interpretation, engagement, and communication between AI and clinicians are crucial to developing practical workflows.

## Conclusions

In response to the emergency of the COVID-19 pandemic, the US Food & Drug Administration (FDA) has used its Emergency Use Authorization (EUA) authority to allow for the use of a medicine or testing device without all the evidence that is normally required. By July 23^rd^, 2021, FDA authorized 395 tests and sample collection kits for SARS-CoV-2 detection under EUAs [226]. Noticeably, 53 of these can be used with home-collected specimens and 11 of these were authorized for at-home test [226], reflecting an unprecedented trend in the diagnostics market. BME has expanded over the border of applied biological and medical sciences, employing the knowledge of interdisciplinary research from the collaborations with mechanical, chemical, physical, and computer engineers, shifting the focus of diagnostic research to POCT and personal testing solutions while upgrading available tools.

In this review, we have summarized one and a half years full of innovations in the field of BME research for COVID-19 diagnostics. While immunoassay-based and NAAT-based diagnostics tools have demonstrated their critical role in our quick response to the initial outbreak, the fast spread and persistence of SARS-CoV-2 have continuously forced the researchers to seek more versatile (iNAATs), precise (CRISPR), high-throughput (deep learning), cost-saving and personalized (microfluidic devices and biosensors) solutions. Nevertheless, none of the single methods is perfect for controlling the disease. Therefore, the development of each method needs to be more specialized in coordinating with the others, much like layers of a Swiss Cheese Model. It is anticipated that in the near future, more and more technology will reach the maturation stage and become essential parts of the new normal in the era of COVID-19. While some BME technologies such as PCR and ELISA seem to have reached their peak of development, iNAATs and other POCT diagnoses will continuously benefit from interdisciplinary research, and they need to focus more on practical perspectives such as cost optimization, portability, versatility, and environmental friendliness. Not only for dealing with this pandemic, but the achievements of BME in this field will provide powerful tools for ensuring health and well-being for all, as a goal for sustainable development that the United Nations established.

## Research highlights

- COVID-19 is one of the most severe global health crises that humanity has ever faced

- Biomedical engineering (BME) has been extensively and intensively applied to diagnose COVID-19

- Immunoassay-based and NAAT-based diagnostics tools play a critical role in quick response to the initial outbreak

- More versatile, precise, high-throughput, and cost-saving solutions are needed for later phases of the pandemic

- But none of the single methods is perfect for controlling the disease.

- More and more BME inventions need to be developed and will become essential parts of the new normal in the era of COVID-19
